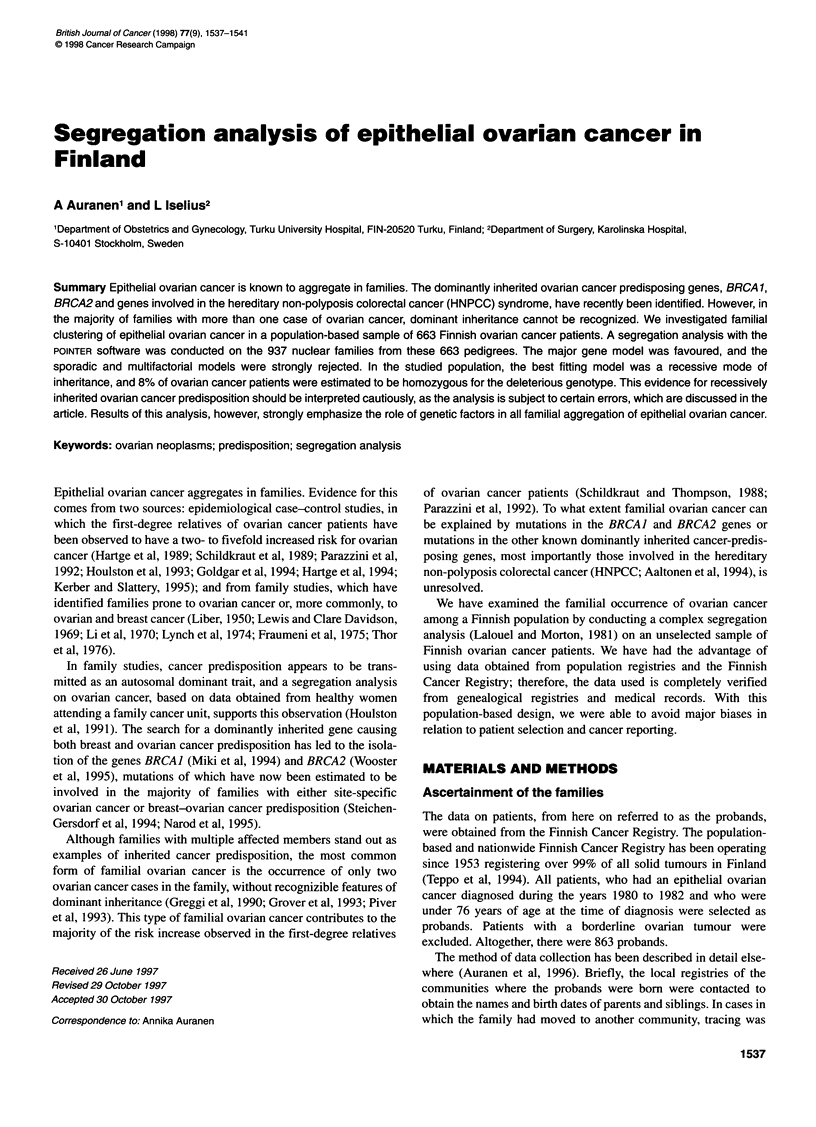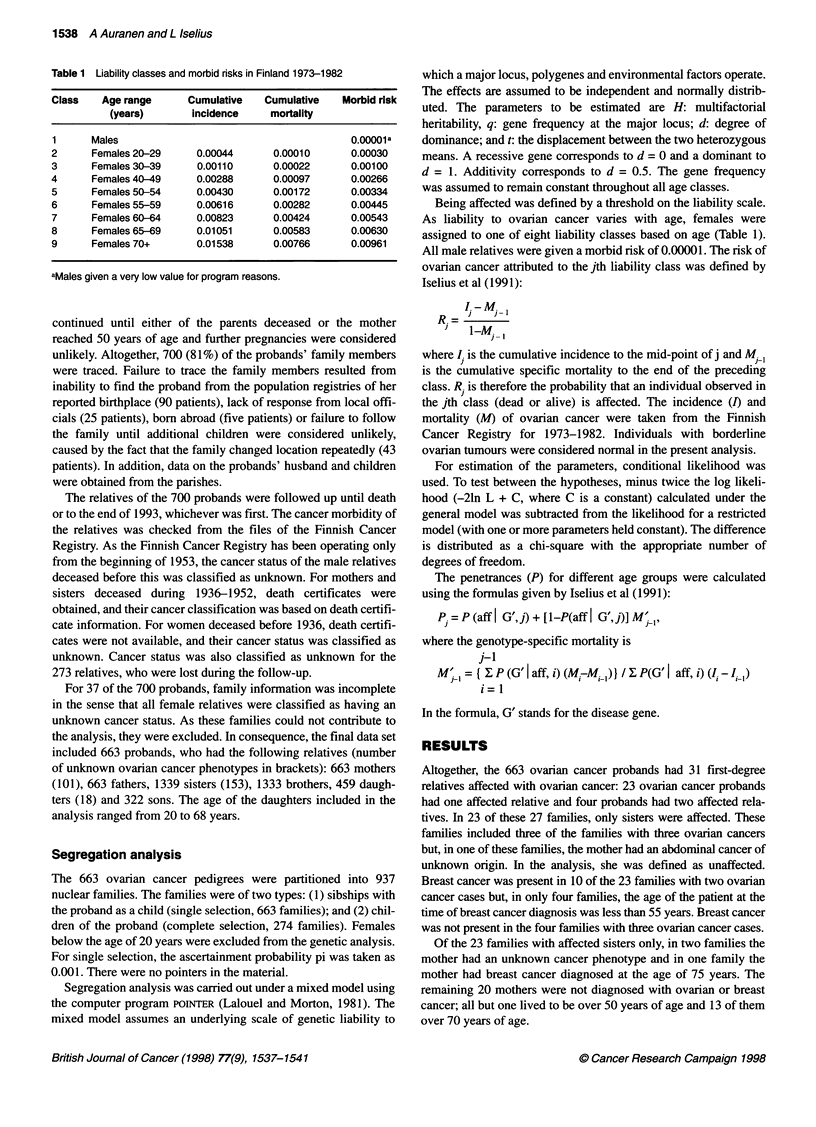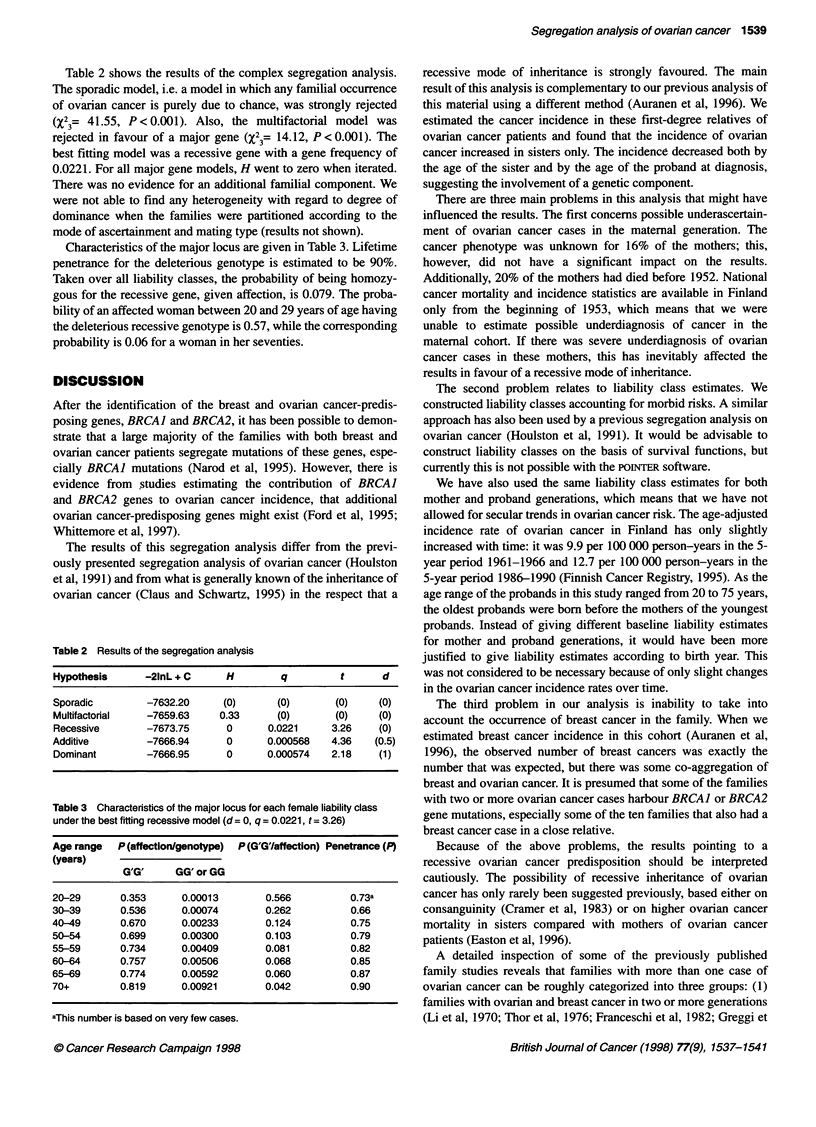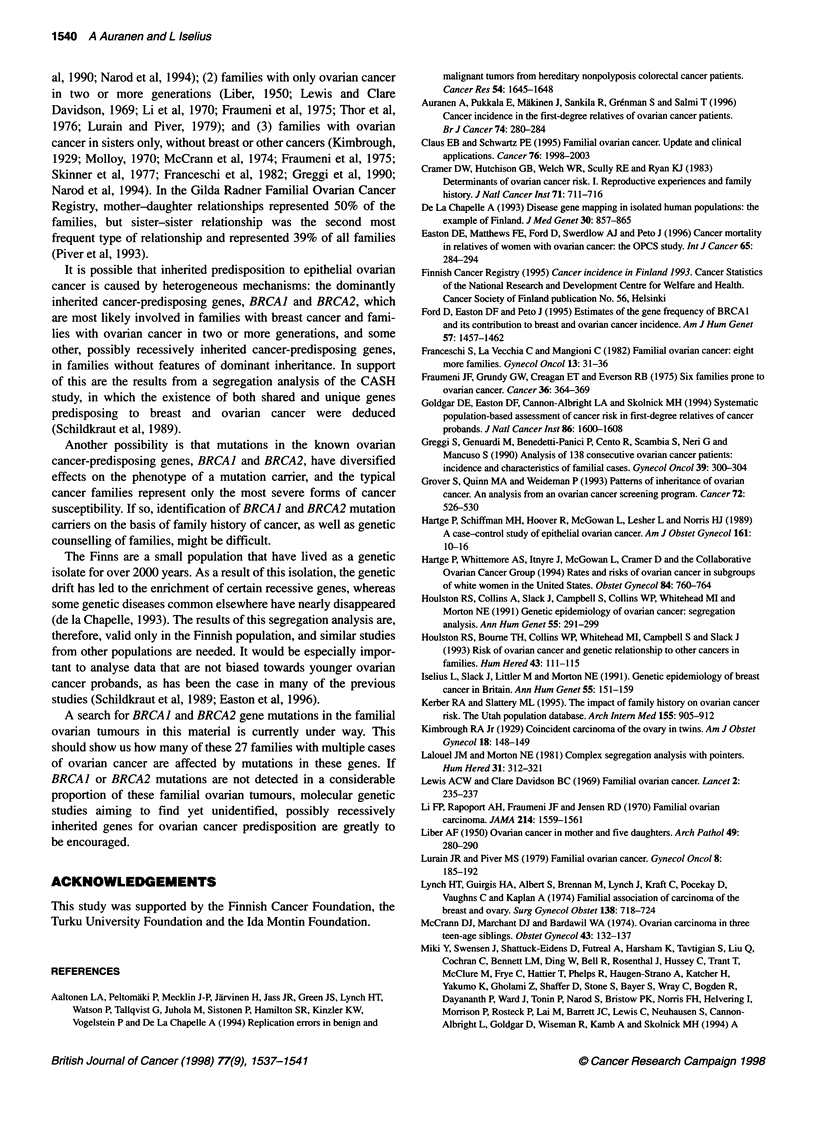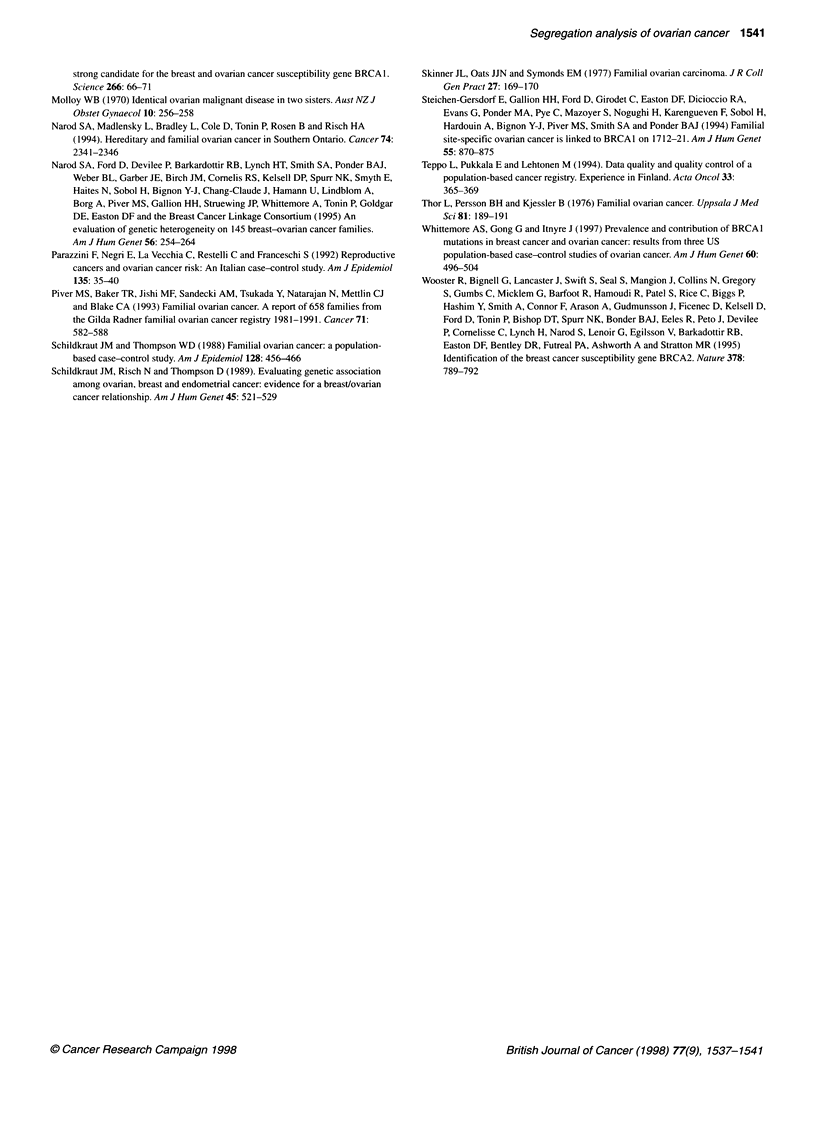# Segregation analysis of epithelial ovarian cancer in Finland.

**DOI:** 10.1038/bjc.1998.252

**Published:** 1998-05

**Authors:** A. Auranen, L. Iselius

**Affiliations:** Department of Obstetrics and Gynecology, Turku University Hospital, Finland.

## Abstract

Epithelial ovarian cancer is known to aggregate in families. The dominantly inherited ovarian cancer predisposing genes, BRCA1, BRCA2 and genes involved in the hereditary non-polyposis colorectal cancer (HNPCC) syndrome, have recently been identified. However, in the majority of families with more than one case of ovarian cancer, dominant inheritance cannot be recognized. We investigated familial clustering of epithelial ovarian cancer in a population-based sample of 663 Finnish ovarian cancer patients. A segregation analysis with the POINTER software was conducted on the 937 nuclear families from these 663 pedigrees. The major gene model was favoured, and the sporadic and multifactorial models were strongly rejected. In the studied population, the best fitting model was a recessive mode of inheritance, and 8% of ovarian cancer patients were estimated to be homozygous for the deleterious genotype. This evidence for recessively inherited ovarian cancer predisposition should be interpreted cautiously, as the analysis is subject to certain errors, which are discussed in the article. Results of this analysis, however, strongly emphasize the role of genetic factors in all familial aggregation of epithelial ovarian cancer.


British Journal of Cancer (1998) 77(9), 1537–1541
© 1998 Cancer Research Campaign

### Segregation analysis of epithelial ovarian cancer in Finland

**A Auranen[1] and L Iselius[2]**

[1]Department of Obstetrics and Gynecology, Turku University Hospital, FIN-20520 Turku, Finland; [2]Department of Surgery, Karolinska Hospital, S-10401 Stockholm, Sweden

**Summary** Epithelial ovarian cancer is known to aggregate in families. The dominantly inherited ovarian cancer predisposing genes, *BRCA1*, *BRCA2* and genes involved in the hereditary non-polyposis colorectal cancer (HNPCC) syndrome, have recently been identified. However, in the majority of families with more than one case of ovarian cancer, dominant inheritance cannot be recognized. We investigated familial clustering of epithelial ovarian cancer in a population-based sample of 663 Finnish ovarian cancer patients. A segregation analysis with the POINTER software was conducted on the 937 nuclear families from these 663 pedigrees. The major gene model was favoured, and the sporadic and multifactorial models were strongly rejected. In the studied population, the best fitting model was a recessive mode of inheritance, and 8% of ovarian cancer patients were estimated to be homozygous for the deleterious genotype. This evidence for recessively inherited ovarian cancer predisposition should be interpreted cautiously, as the analysis is subject to certain errors, which are discussed in the article. Results of this analysis, however, strongly emphasize the role of genetic factors in all familial aggregation of epithelial ovarian cancer.

**Keywords:** ovarian neoplasms; predisposition; segregation analysis

Epithelial ovarian cancer aggregates in families. Evidence for this comes from two sources: epidemiological case–control studies, in which the first-degree relatives of ovarian cancer patients have been observed to have a two- to fivefold increased risk for ovarian cancer (Hartge et al, 1989; Schildkraut et al, 1989; Parazzini et al, 1992; Houlston et al, 1993; Goldgar et al, 1994; Hartge et al, 1994; Kerber and Slattery, 1995); and from family studies, which have identified families prone to ovarian cancer or, more commonly, to ovarian and breast cancer (Liber, 1950; Lewis and Clare Davidson, 1969; Li et al, 1970; Lynch et al, 1974; Fraumeni et al, 1975; Thor et al, 1976).

In family studies, cancer predisposition appears to be transmitted as an autosomal dominant trait, and a segregation analysis on ovarian cancer, based on data obtained from healthy women attending a family cancer unit, supports this observation (Houlston et al, 1991). The search for a dominantly inherited gene causing both breast and ovarian cancer predisposition has led to the isolation of the genes *BRCA1* (Miki et al, 1994) and *BRCA2* (Wooster et al, 1995), mutations of which have now been estimated to be involved in the majority of families with either site-specific ovarian cancer or breast–ovarian cancer predisposition (Steichen-Gersdorf et al, 1994; Narod et al, 1995).

Although families with multiple affected members stand out as examples of inherited cancer predisposition, the most common form of familial ovarian cancer is the occurrence of only two ovarian cancer cases in the family, without recognizible features of dominant inheritance (Greggi et al, 1990; Grover et al, 1993; Piver et al, 1993). This type of familial ovarian cancer contributes to the majority of the risk increase observed in the first-degree relatives of ovarian cancer patients (Schildkraut and Thompson, 1988; Parazzini et al, 1992). To what extent familial ovarian cancer can be explained by mutations in the *BRCA1* and *BRCA2* genes or mutations in the other known dominantly inherited cancer-predisposing genes, most importantly those involved in the hereditary non-polyposis colorectal cancer (HNPCC; Aaltonen et al, 1994), is unresolved.

We have examined the familial occurrence of ovarian cancer among a Finnish population by conducting a complex segregation analysis (Lalouel and Morton, 1981) on an unselected sample of Finnish ovarian cancer patients. We have had the advantage of using data obtained from population registries and the Finnish Cancer Registry; therefore, the data used is completely verified from genealogical registries and medical records. With this population-based design, we were able to avoid major biases in relation to patient selection and cancer reporting.

### MATERIALS AND METHODS

### Ascertainment of the families

The data on patients, from here on referred to as the probands, were obtained from the Finnish Cancer Registry. The population-based and nationwide Finnish Cancer Registry has been operating since 1953 registering over 99% of all solid tumours in Finland (Teppo et al, 1994). All patients, who had an epithelial ovarian cancer diagnosed during the years 1980 to 1982 and who were under 76 years of age at the time of diagnosis were selected as probands. Patients with a borderline ovarian tumour were excluded. Altogether, there were 863 probands.

The method of data collection has been described in detail elsewhere (Auranen et al, 1996). Briefly, the local registries of the communities where the probands were born were contacted to obtain the names and birth dates of parents and siblings. In cases in which the family had moved to another community, tracing was

*Received 26 June 1997*
*Revised 29 October 1997*
*Accepted 30 October 1997*

*Correspondence to:* Annika Auranen

Table 1 Liability classes and morbid risks in Finland 1973–1982

| Class | Age range (years) | Cumulative incidence | Cumulative mortality | Morbid risk |
|---|---|---|---|---|
| 1 | Males | | | 0.00001[a] |
| 2 | Females 20–29 | 0.00044 | 0.00010 | 0.00030 |
| 3 | Females 30–39 | 0.00110 | 0.00022 | 0.00100 |
| 4 | Females 40–49 | 0.00288 | 0.00097 | 0.00266 |
| 5 | Females 50–54 | 0.00430 | 0.00172 | 0.00334 |
| 6 | Females 55–59 | 0.00616 | 0.00282 | 0.00445 |
| 7 | Females 60–64 | 0.00823 | 0.00424 | 0.00543 |
| 8 | Females 65–69 | 0.01051 | 0.00583 | 0.00630 |
| 9 | Females 70+ | 0.01538 | 0.00766 | 0.00961 |

[a]Males given a very low value for program reasons.

continued until either of the parents deceased or the mother reached 50 years of age and further pregnancies were considered unlikely. Altogether, 700 (81%) of the probands' family members were traced. Failure to trace the family members resulted from inability to find the proband from the population registries of her reported birthplace (90 patients), lack of response from local officials (25 patients), born abroad (five patients) or failure to follow the family until additional children were considered unlikely, caused by the fact that the family changed location repeatedly (43 patients). In addition, data on the probands' husband and children were obtained from the parishes.

The relatives of the 700 probands were followed up until death or to the end of 1993, whichever was first. The cancer morbidity of the relatives was checked from the files of the Finnish Cancer Registry. As the Finnish Cancer Registry has been operating only from the beginning of 1953, the cancer status of the male relatives deceased before this was classified as unknown. For mothers and sisters deceased during 1936–1952, death certificates were obtained, and their cancer classification was based on death certificate information. For women deceased before 1936, death certificates were not available, and their cancer status was classified as unknown. Cancer status was also classified as unknown for the 273 relatives, who were lost during the follow-up.

For 37 of the 700 probands, family information was incomplete in the sense that all female relatives were classified as having an unknown cancer status. As these families could not contribute to the analysis, they were excluded. In consequence, the final data set included 663 probands, who had the following relatives (number of unknown ovarian cancer phenotypes in brackets): 663 mothers (101), 663 fathers, 1339 sisters (153), 1333 brothers, 459 daughters (18) and 322 sons. The age of the daughters included in the analysis ranged from 20 to 68 years.

### Segregation analysis

The 663 ovarian cancer pedigrees were partitioned into 937 nuclear families. The families were of two types: (1) sibships with the proband as a child (single selection, 663 families); and (2) children of the proband (complete selection, 274 families). Females below the age of 20 years were excluded from the genetic analysis. For single selection, the ascertainment probability pi was taken as 0.001. There were no pointers in the material.

Segregation analysis was carried out under a mixed model using the computer program POINTER (Lalouel and Morton, 1981). The mixed model assumes an underlying scale of genetic liability to which a major locus, polygenes and environmental factors operate. The effects are assumed to be independent and normally distributed. The parameters to be estimated are *H*: multifactorial heritability, *q*: gene frequency at the major locus; *d*: degree of dominance; and *t*: the displacement between the two heterozygous means. A recessive gene corresponds to $d = 0$ and a dominant to $d = 1$. Additivity corresponds to $d = 0.5$. The gene frequency was assumed to remain constant throughout all age classes.

Being affected was defined by a threshold on the liability scale. As liability to ovarian cancer varies with age, females were assigned to one of eight liability classes based on age (Table 1). All male relatives were given a morbid risk of 0.00001. The risk of ovarian cancer attributed to the *j*th liability class was defined by Iselius et al (1991):

$$R_j = \frac{I_j - M_{j-1}}{1 - M_{j-1}}$$

where $I_j$ is the cumulative incidence to the mid-point of j and $M_{j-1}$ is the cumulative specific mortality to the end of the preceding class. $R_j$ is therefore the probability that an individual observed in the *j*th class (dead or alive) is affected. The incidence (*I*) and mortality (*M*) of ovarian cancer were taken from the Finnish Cancer Registry for 1973–1982. Individuals with borderline ovarian tumours were considered normal in the present analysis.

For estimation of the parameters, conditional likelihood was used. To test between the hypotheses, minus twice the log likelihood (–2ln L + C, where C is a constant) calculated under the general model was subtracted from the likelihood for a restricted model (with one or more parameters held constant). The difference is distributed as a chi-square with the appropriate number of degrees of freedom.

The penetrances (*P*) for different age groups were calculated using the formulas given by Iselius et al (1991):

$$P_j = P(\text{aff} \mid G', j) + [1 - P(\text{aff} \mid G', j)]\, M'_{j-1},$$

where the genotype-specific mortality is

$$M'_{j-1} = \{ \sum_{i=1}^{j-1} P(G' \mid \text{aff}, i)\,(M_i - M_{i-1}) \} / \sum P(G' \mid \text{aff}, i)\,(I_i - I_{i-1})$$

In the formula, G′ stands for the disease gene.

### RESULTS

Altogether, the 663 ovarian cancer probands had 31 first-degree relatives affected with ovarian cancer: 23 ovarian cancer probands had one affected relative and four probands had two affected relatives. In 23 of these 27 families, only sisters were affected. These families included three of the families with three ovarian cancers but, in one of these families, the mother had an abdominal cancer of unknown origin. In the analysis, she was defined as unaffected. Breast cancer was present in 10 of the 23 families with two ovarian cancer cases but, in only four families, the age of the patient at the time of breast cancer diagnosis was less than 55 years. Breast cancer was not present in the four families with three ovarian cancer cases.

Of the 23 families with affected sisters only, in two families the mother had an unknown cancer phenotype and in one family the mother had breast cancer diagnosed at the age of 75 years. The remaining 20 mothers were not diagnosed with ovarian or breast cancer; all but one lived to be over 50 years of age and 13 of them over 70 years of age.

© Cancer Research Campaign 1998

Table 2 shows the results of the complex segregation analysis. The sporadic model, i.e. a model in which any familial occurrence of ovarian cancer is purely due to chance, was strongly rejected ($\chi^2_3$= 41.55, $P < 0.001$). Also, the multifactorial model was rejected in favour of a major gene ($\chi^2_3$= 14.12, $P < 0.001$). The best fitting model was a recessive gene with a gene frequency of 0.0221. For all major gene models, $H$ went to zero when iterated. There was no evidence for an additional familial component. We were not able to find any heterogeneity with regard to degree of dominance when the families were partitioned according to the mode of ascertainment and mating type (results not shown).

Characteristics of the major locus are given in Table 3. Lifetime penetrance for the deleterious genotype is estimated to be 90%. Taken over all liability classes, the probability of being homozygous for the recessive gene, given affection, is 0.079. The probability of an affected woman between 20 and 29 years of age having the deleterious recessive genotype is 0.57, while the corresponding probability is 0.06 for a woman in her seventies.

### DISCUSSION

After the identification of the breast and ovarian cancer-predisposing genes, *BRCA1* and *BRCA2*, it has been possible to demonstrate that a large majority of the families with both breast and ovarian cancer patients segregate mutations of these genes, especially *BRCA1* mutations (Narod et al, 1995). However, there is evidence from studies estimating the contribution of *BRCA1* and *BRCA2* genes to ovarian cancer incidence, that additional ovarian cancer-predisposing genes might exist (Ford et al, 1995; Whittemore et al, 1997).

The results of this segregation analysis differ from the previously presented segregation analysis of ovarian cancer (Houlston et al, 1991) and from what is generally known of the inheritance of ovarian cancer (Claus and Schwartz, 1995) in the respect that a recessive mode of inheritance is strongly favoured. The main result of this analysis is complementary to our previous analysis of this material using a different method (Auranen et al, 1996). We estimated the cancer incidence in these first-degree relatives of ovarian cancer patients and found that the incidence of ovarian cancer increased in sisters only. The incidence decreased both by the age of the sister and by the age of the proband at diagnosis, suggesting the involvement of a genetic component.

There are three main problems in this analysis that might have influenced the results. The first concerns possible underascertainment of ovarian cancer cases in the maternal generation. The cancer phenotype was unknown for 16% of the mothers; this, however, did not have a significant impact on the results. Additionally, 20% of the mothers had died before 1952. National cancer mortality and incidence statistics are available in Finland only from the beginning of 1953, which means that we were unable to estimate possible underdiagnosis of cancer in the maternal cohort. If there was severe underdiagnosis of ovarian cancer cases in these mothers, this has inevitably affected the results in favour of a recessive mode of inheritance.

The second problem relates to liability class estimates. We constructed liability classes accounting for morbid risks. A similar approach has also been used by a previous segregation analysis on ovarian cancer (Houlston et al, 1991). It would be advisable to construct liability classes on the basis of survival functions, but currently this is not possible with the POINTER software.

We have also used the same liability class estimates for both mother and proband generations, which means that we have not allowed for secular trends in ovarian cancer risk. The age-adjusted incidence rate of ovarian cancer in Finland has only slightly increased with time: it was 9.9 per 100 000 person–years in the 5-year period 1961–1966 and 12.7 per 100 000 person–years in the 5-year period 1986–1990 (Finnish Cancer Registry, 1995). As the age range of the probands in this study ranged from 20 to 75 years, the oldest probands were born before the mothers of the youngest probands. Instead of giving different baseline liability estimates for mother and proband generations, it would have been more justified to give liability estimates according to birth year. This was not considered to be necessary because of only slight changes in the ovarian cancer incidence rates over time.

The third problem in our analysis is inability to take into account the occurrence of breast cancer in the family. When we estimated breast cancer incidence in this cohort (Auranen et al, 1996), the observed number of breast cancers was exactly the number that was expected, but there was some co-aggregation of breast and ovarian cancer. It is presumed that some of the families with two or more ovarian cancer cases harbour *BRCA1* or *BRCA2* gene mutations, especially some of the ten families that also had a breast cancer case in a close relative.

Because of the above problems, the results pointing to a recessive ovarian cancer predisposition should be interpreted cautiously. The possibility of recessive inheritance of ovarian cancer has only rarely been suggested previously, based either on consanguinity (Cramer et al, 1983) or on higher ovarian cancer mortality in sisters compared with mothers of ovarian cancer patients (Easton et al, 1996).

A detailed inspection of some of the previously published family studies reveals that families with more than one case of ovarian cancer can be roughly categorized into three groups: (1) families with ovarian and breast cancer in two or more generations (Li et al, 1970; Thor et al, 1976; Franceschi et al, 1982; Greggi et

**Table 2** Results of the segregation analysis

| Hypothesis | −2lnL + C | *H* | *q* | *t* | *d* |
|---|---|---|---|---|---|
| Sporadic | −7632.20 | (0) | (0) | (0) | (0) |
| Multifactorial | −7659.63 | 0.33 | (0) | (0) | (0) |
| Recessive | −7673.75 | 0 | 0.0221 | 3.26 | (0) |
| Additive | −7666.94 | 0 | 0.000568 | 4.36 | (0.5) |
| Dominant | −7666.95 | 0 | 0.000574 | 2.18 | (1) |

**Table 3** Characteristics of the major locus for each female liability class under the best fitting recessive model ($d$ = 0, $q$ = 0.0221, $t$ = 3.26)

| Age range (years) | *P* (affection/genotype) | | *P* (G′G′/affection) | Penetrance (*P*) |
|---|---|---|---|---|
| | G′G′ | GG′ or GG | | |
| 20–29 | 0.353 | 0.00013 | 0.566 | 0.73[a] |
| 30–39 | 0.536 | 0.00074 | 0.262 | 0.66 |
| 40–49 | 0.670 | 0.00233 | 0.124 | 0.75 |
| 50–54 | 0.699 | 0.00300 | 0.103 | 0.79 |
| 55–59 | 0.734 | 0.00409 | 0.081 | 0.82 |
| 60–64 | 0.757 | 0.00506 | 0.068 | 0.85 |
| 65–69 | 0.774 | 0.00592 | 0.060 | 0.87 |
| 70+ | 0.819 | 0.00921 | 0.042 | 0.90 |

[a]This number is based on very few cases.

© Cancer Research Campaign 1998

al, 1990; Narod et al, 1994); (2) families with only ovarian cancer in two or more generations (Liber, 1950; Lewis and Clare Davidson, 1969; Li et al, 1970; Fraumeni et al, 1975; Thor et al, 1976; Lurain and Piver, 1979); and (3) families with ovarian cancer in sisters only, without breast or other cancers (Kimbrough, 1929; Molloy, 1970; McCrann et al, 1974; Fraumeni et al, 1975; Skinner et al, 1977; Franceschi et al, 1982; Greggi et al, 1990; Narod et al, 1994). In the Gilda Radner Familial Ovarian Cancer Registry, mother–daughter relationships represented 50% of the families, but sister–sister relationship was the second most frequent type of relationship and represented 39% of all families (Piver et al, 1993).

It is possible that inherited predisposition to epithelial ovarian cancer is caused by heterogeneous mechanisms: the dominantly inherited cancer-predisposing genes, *BRCA1* and *BRCA2*, which are most likely involved in families with breast cancer and families with ovarian cancer in two or more generations, and some other, possibly recessively inherited cancer-predisposing genes, in families without features of dominant inheritance. In support of this are the results from a segregation analysis of the CASH study, in which the existence of both shared and unique genes predisposing to breast and ovarian cancer were deduced (Schildkraut et al, 1989).

Another possibility is that mutations in the known ovarian cancer-predisposing genes, *BRCA1* and *BRCA2*, have diversified effects on the phenotype of a mutation carrier, and the typical cancer families represent only the most severe forms of cancer susceptibility. If so, identification of *BRCA1* and *BRCA2* mutation carriers on the basis of family history of cancer, as well as genetic counselling of families, might be difficult.

The Finns are a small population that have lived as a genetic isolate for over 2000 years. As a result of this isolation, the genetic drift has led to the enrichment of certain recessive genes, whereas some genetic diseases common elsewhere have nearly disappeared (de la Chapelle, 1993). The results of this segregation analysis are, therefore, valid only in the Finnish population, and similar studies from other populations are needed. It would be especially important to analyse data that are not biased towards younger ovarian cancer probands, as has been the case in many of the previous studies (Schildkraut et al, 1989; Easton et al, 1996).

A search for *BRCA1* and *BRCA2* gene mutations in the familial ovarian tumours in this material is currently under way. This should show us how many of these 27 families with multiple cases of ovarian cancer are affected by mutations in these genes. If *BRCA1* or *BRCA2* mutations are not detected in a considerable proportion of these familial ovarian tumours, molecular genetic studies aiming to find yet unidentified, possibly recessively inherited genes for ovarian cancer predisposition are greatly to be encouraged.

### ACKNOWLEDGEMENTS

This study was supported by the Finnish Cancer Foundation, the Turku University Foundation and the Ida Montin Foundation.

### REFERENCES

Aaltonen LA, Peltomäki P, Mecklin J-P, Järvinen H, Jass JR, Green JS, Lynch HT, Watson P, Tallqvist G, Juhola M, Sistonen P, Hamilton SR, Kinzler KW, Vogelstein P and De La Chapelle A (1994) Replication errors in benign and malignant tumors from hereditary nonpolyposis colorectal cancer patients. *Cancer Res* **54**: 1645–1648

Auranen A, Pukkala E, Mäkinen J, Sankila R, Grénman S and Salmi T (1996) Cancer incidence in the first-degree relatives of ovarian cancer patients. *Br J Cancer* **74**: 280–284

Claus EB and Schwartz PE (1995) Familial ovarian cancer. Update and clinical applications. *Cancer* **76**: 1998–2003

Cramer DW, Hutchison GB, Welch WR, Scully RE and Ryan KJ (1983) Determinants of ovarian cancer risk. I. Reproductive experiences and family history. *J Natl Cancer Inst* **71**: 711–716

De La Chapelle A (1993) Disease gene mapping in isolated human populations: the example of Finland. *J Med Genet* **30**: 857–865

Easton DE, Matthews FE, Ford D, Swerdlow AJ and Peto J (1996) Cancer mortality in relatives of women with ovarian cancer: the OPCS study. *Int J Cancer* **65**: 284–294

Finnish Cancer Registry (1995) *Cancer incidence in Finland 1993*. Cancer Statistics of the National Research and Development Centre for Welfare and Health. Cancer Society of Finland publication No. 56, Helsinki

Ford D, Easton DF and Peto J (1995) Estimates of the gene frequency of BRCA1 and its contribution to breast and ovarian cancer incidence. *Am J Hum Genet* **57**: 1457–1462

Franceschi S, La Vecchia C and Mangioni C (1982) Familial ovarian cancer: eight more families. *Gynecol Oncol* **13**: 31–36

Fraumeni JF, Grundy GW, Creagan ET and Everson RB (1975) Six families prone to ovarian cancer. *Cancer* **36**: 364–369

Goldgar DE, Easton DF, Cannon-Albright LA and Skolnick MH (1994) Systematic population-based assessment of cancer risk in first-degree relatives of cancer probands. *J Natl Cancer Inst* **86**: 1600–1608

Greggi S, Genuardi M, Benedetti-Panici P, Cento R, Scambia S, Neri G and Mancuso S (1990) Analysis of 138 consecutive ovarian cancer patients: incidence and characteristics of familial cases. *Gynecol Oncol* **39**: 300–304

Grover S, Quinn MA and Weideman P (1993) Patterns of inheritance of ovarian cancer. An analysis from an ovarian cancer screening program. *Cancer* **72**: 526–530

Hartge P, Schiffman MH, Hoover R, McGowan L, Lesher L and Norris HJ (1989) A case–control study of epithelial ovarian cancer. *Am J Obstet Gynecol* **161**: 10–16

Hartge P, Whittemore AS, Itnyre J, McGowan L, Cramer D and the Collaborative Ovarian Cancer Group (1994) Rates and risks of ovarian cancer in subgroups of white women in the United States. *Obstet Gynecol* **84**: 760–764

Houlston RS, Collins A, Slack J, Campbell S, Collins WP, Whitehead MI and Morton NE (1991) Genetic epidemiology of ovarian cancer: segregation analysis. *Ann Hum Genet* **55**: 291–299

Houlston RS, Bourne TH, Collins WP, Whitehead MI, Campbell S and Slack J (1993) Risk of ovarian cancer and genetic relationship to other cancers in families. *Hum Hered* **43**: 111–115

Iselius L, Slack J, Littler M and Morton NE (1991). Genetic epidemiology of breast cancer in Britain. *Ann Hum Genet* **55**: 151–159

Kerber RA and Slattery ML (1995). The impact of family history on ovarian cancer risk. The Utah population database. *Arch Intern Med* **155**: 905–912

Kimbrough RA Jr (1929) Coincident carcinoma of the ovary in twins. *Am J Obstet Gynecol* **18**: 148–149

Lalouel JM and Morton NE (1981) Complex segregation analysis with pointers. *Hum Hered* **31**: 312–321

Lewis ACW and Clare Davidson BC (1969) Familial ovarian cancer. *Lancet* **2**: 235–237

Li FP, Rapoport AH, Fraumeni JF and Jensen RD (1970) Familial ovarian carcinoma. *JAMA* **214**: 1559–1561

Liber AF (1950) Ovarian cancer in mother and five daughters. *Arch Pathol* **49**: 280–290

Lurain JR and Piver MS (1979) Familial ovarian cancer. *Gynecol Oncol* **8**: 185–192

Lynch HT, Guirgis HA, Albert S, Brennan M, Lynch J, Kraft C, Pocekay D, Vaughns C and Kaplan A (1974) Familial association of carcinoma of the breast and ovary. *Surg Gynecol Obstet* **138**: 718–724

McCrann DJ, Marchant DJ and Bardawil WA (1974). Ovarian carcinoma in three teen-age siblings. *Obstet Gynecol* **43**: 132–137

Miki Y, Swensen J, Shattuck-Eidens D, Futreal A, Harsham K, Tavtigian S, Liu Q, Cochran C, Bennett LM, Ding W, Bell R, Rosenthal J, Hussey C, Trant T, McClure M, Frye C, Hattier T, Phelps R, Haugen-Strano A, Katcher H, Yakumo K, Gholami Z, Shaffer D, Stone S, Bayer S, Wray C, Bogden R, Dayananth P, Ward J, Tonin P, Narod S, Bristow PK, Norris FH, Helvering I, Morrison P, Rosteck P, Lai M, Barrett JC, Lewis C, Neuhausen S, Cannon-Albright L, Goldgar D, Wiseman R, Kamb A and Skolnick MH (1994) A

© Cancer Research Campaign 1998

strong candidate for the breast and ovarian cancer susceptibility gene BRCA1. *Science* **266**: 66–71

Molloy WB (1970) Identical ovarian malignant disease in two sisters. *Aust NZ J Obstet Gynaecol* **10**: 256–258

Narod SA, Madlensky L, Bradley L, Cole D, Tonin P, Rosen B and Risch HA (1994). Hereditary and familial ovarian cancer in Southern Ontario. *Cancer* **74**: 2341–2346

Narod SA, Ford D, Devilee P, Barkardottir RB, Lynch HT, Smith SA, Ponder BAJ, Weber BL, Garber JE, Birch JM, Cornelis RS, Kelsell DP, Spurr NK, Smyth E, Haites N, Sobol H, Bignon Y-J, Chang-Claude J, Hamann U, Lindblom A, Borg A, Piver MS, Gallion HH, Struewing JP, Whittemore A, Tonin P, Goldgar DE, Easton DF and the Breast Cancer Linkage Consortium (1995) An evaluation of genetic heterogeneity on 145 breast–ovarian cancer families. *Am J Hum Genet* **56**: 254–264

Parazzini F, Negri E, La Vecchia C, Restelli C and Franceschi S (1992) Reproductive cancers and ovarian cancer risk: An Italian case–control study. *Am J Epidemiol* **135**: 35–40

Piver MS, Baker TR, Jishi MF, Sandecki AM, Tsukada Y, Natarajan N, Mettlin CJ and Blake CA (1993) Familial ovarian cancer. A report of 658 families from the Gilda Radner familial ovarian cancer registry 1981–1991. *Cancer* **71**: 582–588

Schildkraut JM and Thompson WD (1988) Familial ovarian cancer: a population-based case–control study. *Am J Epidemiol* **128**: 456–466

Schildkraut JM, Risch N and Thompson D (1989). Evaluating genetic association among ovarian, breast and endometrial cancer: evidence for a breast/ovarian cancer relationship. *Am J Hum Genet* **45**: 521–529

Skinner JL, Oats JJN and Symonds EM (1977) Familial ovarian carcinoma. *J R Coll Gen Pract* **27**: 169–170

Steichen-Gersdorf E, Gallion HH, Ford D, Girodet C, Easton DF, Dicioccio RA, Evans G, Ponder MA, Pye C, Mazoyer S, Nogughi H, Karengueven F, Sobol H, Hardouin A, Bignon Y-J, Piver MS, Smith SA and Ponder BAJ (1994) Familial site-specific ovarian cancer is linked to BRCA1 on 1712–21. *Am J Hum Genet* **55**: 870–875

Teppo L, Pukkala E and Lehtonen M (1994). Data quality and quality control of a population-based cancer registry. Experience in Finland. *Acta Oncol* **33**: 365–369

Thor L, Persson BH and Kjessler B (1976) Familial ovarian cancer. *Uppsala J Med Sci* **81**: 189–191

Whittemore AS, Gong G and Itnyre J (1997) Prevalence and contribution of BRCA1 mutations in breast cancer and ovarian cancer: results from three US population-based case–control studies of ovarian cancer. *Am J Hum Genet* **60**: 496–504

Wooster R, Bignell G, Lancaster J, Swift S, Seal S, Mangion J, Collins N, Gregory S, Gumbs C, Micklem G, Barfoot R, Hamoudi R, Patel S, Rice C, Biggs P, Hashim Y, Smith A, Connor F, Arason A, Gudmunsson J, Ficenec D, Kelsell D, Ford D, Tonin P, Bishop DT, Spurr NK, Bonder BAJ, Eeles R, Peto J, Devilee P, Cornelisse C, Lynch H, Narod S, Lenoir G, Egilsson V, Barkadottir RB, Easton DF, Bentley DR, Futreal PA, Ashworth A and Stratton MR (1995) Identification of the breast cancer susceptibility gene BRCA2. *Nature* **378**: 789–792

© Cancer Research Campaign 1998